# Risk Factors for Breast Cancer among Jordanian Women: A Case-control Study

**Published:** 2018-01

**Authors:** Mohammad AL QADIRE, Murad ALKHALAILEH, Hedaya HINA

**Affiliations:** 1. Dept. of Adult Health, Nursing School, Al al-Bayt University, Mafraq, Jordan; 2. Dept. of Nursing, Tabuk University, Tabuk, Saudi Arabia; 3. King Hussein Cancer Center, Amman, Jordan

**Keywords:** Lifestyle, Breast cancer, Risk factors, Dietary, Physical activity, Jordan

## Abstract

**Background:**

The interaction between inherited mutated genes and environmental factors is believed to play a crucial role in cancer development. The main aim was to identify lifestyle-related risk factors for breast cancer among Jordanian women.

**Methods:**

A hospital-based multicenter case-control study was conducted in Jordan in 2016. Overall, 405 cases and 418 controls, in 3 large hospitals where cancer patients are treated, participated. The prevalence of individual and groups of cancer-related risk factors was estimated descriptively using percentages and odd ratios with their correlated 95% Confidence interval (CI). The predictors of the occurrence of breast cancer were determined using logistic regression to estimate unadjusted association and adjusted association.

**Results:**

Women in the case group (mean=49.2 yr, SD 10.2) were older than those in the control group (mean=45.9, SD 10.9). Physical activity (sufficiently active) (OR=2.76; 95% CI=1.96–3.87) and fruit and vegetable intake (good or optimal) (OR=1.71 95% CI=1.25–2.35) were found to be associated with reduced breast cancer risk. However, calcium intake (>3 times a week) (OR=0.51; 95% CI=0.34–0.77) was associated with increased risk of breast cancer.

**Conclusion:**

Lifestyle risk factors were identified, and certain modifications to lifestyle are needed. Women’s awareness of these factors should be raised through appropriate channels, as a priority of the health authorities. Increasing the amount of high-quality research in this area remains one of the best ways to fight breast cancer, reducing its incidence and associated morbidities.

## Introduction

The interaction between inherited mutated genes and environmental factors is believed to play a crucial role in cancer development (1, 2). Based on this fact, in theory modifying dietary and lifestyle habits might minimize or at least reduce cancer risk factors (1). About 35% of cancer-related deaths are due to modifiable factors such as smoking, alcohol consumption, low fruit and vegetable intake and a high fat-diet (1, 3). From an economic point of view, cancer prevention and early detection measures would reduce the cost of medical care provided for cancer patients (4–6). With regard to breast cancer, lifestyle and diet-related factors may affect (positively or negatively) the likelihood of breast cancer. Women who regularly take intense to moderate exercise, consume a diet rich in fiber, fruit, and vegetables, are not obese, and are non-smokers have a reduced risk of developing breast cancer in their life trajectory (7–10). For example, in a systematic review and meta-analysis to identify the risk factors for breast cancer (11), a comprehensive review of databases was conducted, and 66 studies were included in the final review and meta-analysis. Alcohol intake, having a second-degree relative with breast cancer, and a history of breast biopsy was found to be associated with an increased risk of breast cancer (11).

Total fruit and vegetable intake were associated with a reduction in the risk of breast cancer (10). A high intake of soya bean and red meat reduced the risk of breast cancer among Chinese women (9). Intense and moderate physical activity was also found to reduce the risk (12). However, the various findings are sometimes contradictory. The studies were conducted in different countries, representing different geographical regions and different cultures, which could themselves affect the risk factors. Despite their importance, these studies are limited in developing countries such as Jordan. Thus, a similar study is needed in Jordan in particular and the region in general. This study is the first of its kind in this country and is considered as complementary to international research. The main aim was to identify lifestyle-related risk factors for breast cancer among Jordanian women.

This study aimed to answer the following specific research questions:

1. What are the reproductive risk factors for breast cancer in Jordan related to dietary and lifestyle habits?
2. What are the predictors of breast cancer occurrence among Jordanian women?

## Methods

### Design

A hospital-based multicenter case-control study design was used to explore and identify risk factors for breast cancer.

### Sample

The sample comprised two groups: case and control. Overall, 405 cases and 418 controls participated in the study that was conducted 2016. The case group comprised cancer patients who met the following inclusion criteria: 18 yr or older, confirmed breast cancer diagnosis as evident from histopathology reports, newly diagnosed within six months, knowing their cancer diagnosis, and physically able to complete a 15–30-min structured interview. The control group participants had no cancer and match the participants in the case group with regard to age (±5 yr), gender, and geographical area.

### Sample size

The sample size was calculated based on the number of independent variables (m) in the logistic regression model. For medium-sized relationships between the independent variables and the dependent or outcome variable, 5% significance and 80% power, the sample size (N) should satisfy the following (13): N ≥ 10m (m= number of independent variables). It is planned to allow up to 30 independent variables (based on previous studies) in the regression model, and allowing for a 20% attrition rate N should be at least 360 for each group. A total number of 400 participants in each group were considered adequate.

### Instruments

#### Demographic Data Sheet (DDS)

The DDS includes questions designed to elicit information about participants’ demographic characteristics: age, marital status, education, income, smoking, previous history of cancer. It also collects information about the patients’ general health, and on cancer diagnosis (confirmed histopathology report), stage, and other chronic medical conditions.

#### Anthropometric measures

Height and weight were measured for all participants and the body mass index (BMI) calculated: weight (Kg)/Height (m^2^). Participants with BMI scores 18 to 25 were considered normal, but BMI scores from 25 to 29.9 were considered over-weight and of 30 and above as obese.

### Lifestyle characteristics

Lifestyle characteristics were assessed with regard to physical activity, smoking, and dietary intake. Physical activity was assessed using the brief Physical Activity Questionnaire (14) in which participants were asked about their activities (vigorous or moderate) and accordingly categorized into two levels: sufficiently or insufficiently active. The validity coefficient was 0.40 with a 95% confidence interval (CI) 0.12 to 0.69, confirming the questionnaire as a valid and reliable tool (14). Smoking was assessed by whether participants currently smoke or not, and smokers were asked to quantify the number of cigarettes they smoke a day.

### Dietary intake assessment

Dietary habits were assessed over the previous year using two short questionnaires, for fat and fruit/vegetable intake (15). These tools found to valid when compared with full-length Food Frequency Questionnaire, r=0.61 for the fat screener and r=0.71 for fruit-vegetable screener (15).

***Dietary fat screener:*** this is a self-reported questionnaire of 17 items, asking participants to rate their fat intake. Each item is rated as follows: 0 = once per month or less, 1 = 2–3times a month, 2 =1–2 times a week, 3 = 3–4 times a week, and 4 = 5+ times a week. The total is calculated and classified as one of four categories: 0–7 = very lowfat intake, 8–14 = low or appropriate fat intake, 15–22 = high fat and 23 or more = very high fat intake. For the purpose of analysis, we merged these into two categories: low or appropriate, and high or very high intake.

***Fruit-vegetable screener:*** this is a self-reported questionnaire of 7 items, asking participants to rate their fruit and vegetable intake. Each item is rated as follows: 0 = less than once a week, 1 = once a week, 2 = 2–3 times a week, 3 = 4–6 times a week, 4 = once a day, and 5 = twice or more a day. Then scores are again classified into four categories: 0–10 = very low fruit and vegetable intake, 11–12 = low intake, 13–15 = good intake, and 16 or more = optimal intake. Again, we merged them into two categories: low, and good or optimal intake. These two tools were translated into Arabic using a back-translation approach, and some items were modified to suit the Jordanian population and food habits. Additional questions asked subjects about specific dietary intakes, such as tomatoes, garlic, dairy derivatives, calcium supplement and alcohol.

### Settings

This study was conducted in three large hospitals where most of cancer patients are treated. The first is King Hussein Cancer Center where most of the country’s cancer patients are treated, the second is Al-basheer which is a government referral hospital and the third is King Abdullah University Hospital which is in the north of Jordan.

### Procedure

Ethical approval from Al Al-Bayt university and the appropriate authorities in the selected hospitals was sought. Research assistants then visited each hospital every two weeks over a six-month period, locating all admitted breast cancer patients with the help of the charge nurses and consultants. The study purpose, procedure, and requirements were explained to the patients. If they agreed to participate, they were asked to complete the questionnaire and signed the study consent form in the presence of a research assistant to provide help if needed. The research assistants measured the patient’s weight and height.

The next step was to identify one control participant for each case participant, of the same age (± 5), gender (female only), and geographical area, and having no known cancer. Patients in the case group were asked to suggest a possible control subject (friend, schoolmate or family member); if this was not possible, the research assistant recruited controls from the visitors to other outpatient clinics. Participants in the control group were asked to sign the consent form then to complete the study questionnaire.

### Data analysis

Data was entered into SPSS (version 22, Chicago, IL, USA). The prevalence of individual and groups of cancer-related risk factors was estimated descriptively using percentages, with 95% CI, across the entire sample and for key sub-groups. The statistical association of the presence of individual or groups of risk factors (yes/no) was analyzed using a two-group test, Pearson’s chi-square test for nominal variables. Predictors of the occurrence of breast cancer were determined using logistic regression to estimate unadjusted association (predictors considered separately) and adjusted association (predictors considered together) (16). Finally, odds ratios (OR) and their correlated 95% CI were calculated.

## Results

Women in the case group (mean = 49.2 yr, SD 10.2) were older than those in the control group (mean = 45.9, SD 10.9). There was a significantly higher percentage of highly educated women in the case group (41.7%) than in control group (31.8%) (*P*=0.004). Most of the women in both groups were married (case: 90.1%), control (93.5%) (Table 1).

**Table 1:** Socio-demographic risk factors among women in control and case groups

***Variable***	***Controls (n = 418)***	***Cases (n =405)***	***OR (95% CI)***	**P-*value^a^***

	**n (%)**	**n (%)**		
**Education Level**				
High (≥ diploma)	133 (31.8)	169 (41.7)	1.54 (1.16 to 2.04)	.004^b^
Low (≤ secondary school))	285 (68.2)	236 (58.3)		
**Marital Status**				
Married	391 (93.5)	365 (90.1)	0.63 (0.38 to 1.04)	.076
Never married	27 (6.5)	40 (9.9)		
**Family monthly income**				
Low (< 845$)	344 (82.3)	322 (79.5)	1.19 (0.85 to 1.70)	.330
High (≥ 845$)	74 (17.7)	83 (20.5)		
**Family history of cancer (first-degree relatives)**				
Yes	211 (50.5)	222 (54.8)	1.20 (0.91 to 1.56)	.235
No	207 (49.5)	183 (45.2)		
**Having first-degree relatives with breast cancer**				
Yes	81 (39.1)	107 (48.9)	1.49 (1.01 to 2.18)	.003^b^
No (other types)	126 (60.9)	112 (51.1)		
**Having Diabetes?**				
Yes	54 (12.9)	62 (15.3)	1.21 (0.82 to 1.81)	.367
No	364 (87.1)	343 (84.7)		
**Having Hypertension?**				
Yes	80 (19.1)	100 (24.7)	1.39 (0.99 to 1.93)	.063
No	338 (80.9)	305 (75.3)		
**Body Mass Index (BMI)**				
Normal (≤ 25)	102 (24.4)	88 (21.7)	1.06 (.76 to 1.49)	.733
Overweight or obese (>25)	316 (75.6)	317 (78.3)		
**Working status**				
Yes	347 (83)	310 (76.5)	1.49 (1.06 to 2.11)	.024^b^
No	71 (17)	95 (23.5)		
**Fat intake**				
Low or appropriate	174 (41.6)	168 (41.5)	1.01 (0.76 to 1.33)	.966
High or very high	244 (58.4)	237 (58.5)		
**Fruits and vegetable intake**				
Low	281(67.2)	301 (74.3)	0.79 (0.53 to 0.96)	.025^b^
Good or optimal	137 (32.8)	104 (25.7)		
**Physical activity**				
Insufficient	248 (59.3)	331(81.7)	0.33 (0.24 to 0.45)	.000^b^
Sufficient	170 (40.7)	74 (18.3)		
**Diary intake**				
≤ 2 times a week	151 (36.1%)	149 (29.9)	0.97 (0.73 to 1.29)	.885
> 3 times a week	267 (63.9)	256 (63.2)		
**Tomatoes intake**				
≤ 2 times a week	141 (33.7)	121 (36.8)	1.20 (0.89 to 1.60)	.262
> 3 times a week	277 (66.3)	284 (70.1)		
**Garlic intake**				
≤ 2 times a week	197 (47.1)	203 (50.1)	.88 (0.68 to 1.16)	.403
> 3 times a week	221 (52.9)	202 (49.9)		
**Alcohol intake**				
No	412 (98.6)	397 (98)	0.73 (0.53 to 1.02)	.075
Yes	6 (1.4)	8 (1.9)		
**Practising Breast Self-exam**				
No	332 (79.4)	284 (70.1)	1.65 (1.19 to 2.27)	.002^b^
Yes	86 (20.6)	121 (29.9)		
**Calcium supplement intake**				
≤ 2 times a week	372 (89)	320 (79.0)	2.15 (1.45 to 3.17)	.000^b^
> 3 times a week	46 (87.1)	85 (21.0)		
**Smoking**				
Smoker	70 (16.7)	84 (20.7)	1.30 (0.92 to 1.45)	1.53
Non-smoker	348 (83.3)	321 (79.3)		

a Chi-squared tests comparing control and case groups / /

b Significant: *P*≤ .005

Table 1 shows the results of the chi-squared comparisons, odds ratios (OR), and the 95% CI between the case and control groups with regard to lifestyle-related risk factors. In general, there were no statistically significant associations between marital status, family monthly income, having hypertension, BMI, total fat intake, total dairy intake, tomatoes intake, garlic intake, alcohol intake and smoking. All associated *P*-values were >0.05. However, increased intake of vegetables and fruit was associated with reduced risk of breast cancer (OR=0.79; 95% CI= 0.53–0.96; *P*=0.024). In addition, physical activity was inversely associated with the risk of breast cancer (OR = 0.33; 95% CI = 0.24–0.45 for the women with sufficient activity compared with the insufficiently active women; *P*<0.001). An increased risk of breast cancer by 2.15 times was observed among women with a high total calcium intake, (>3 times a week) (95% CI = 1.45–3.17, *P*<0.001). Finally, the percentage of women practicing self-breast examination was significantly higher in the case group (29.9%) than the control group (20.6%) (OR=1.65; 95% CI =1.19–2.27; *P*=0.002).

All significant factors were further analyzed using a logistic regression model to conclude which factors could be the predictors of breast cancer occurrence (Table 2). Of these, physical activity (sufficiently active) (OR= 2.76; 95% CI= 1.96–3.87) and fruit and vegetable intake (good or optimal) (OR= 1.71 95% CI= 1.25–2.35) were found to be associated with reduced breast cancer risk. However, calcium intake (>3 times a week) (OR= 0.51; 95% CI=0.34–0.77) and breast cancer self-examination (OR 0.59; 95% CI 0.42–0.83) were associated with increased risk of breast cancer.

**Table 2:** Logistic regression model for lifestyle-related risks factors associated with breast cancer

***Risk Factor***	***B***	***S.E.***	**P-*value***	***Odds Ratio***	***95% CI for Odds Ratio***	
Physical activity	1.01	.17	.000	2.75	1.96	3.87
Practicing Breast Self-exam	−0.53	.17	.002	.59	.42	.83
Calcium supplement intake	−0.67	.21	.001	.51	.342	.77
Fruits and vegetables intake	0.54	.16	.001	1.71	1.24	2.35

## Discussion

The main findings of the study suggest the following: sufficient physical activity and optimal total fruit and vegetable intake are protective against breast cancer, high total intake of calcium supplement is a risk factor for breast cancer, and lifestyle-related risk factors vary from one geographical area to another and from culture to culture. Sufficient physical activity and optimal total fruit and vegetable intake are protective against breast cancer; this result supports previously published studies (10, 17–19). The results from 20 studies were analyzed and conducted pooled analysis to examine the association between high intake fruit and vegetable and the risk of breast cancer (10). In these 20 studies, data from 993466 women followed up for 11 to 20 yr were analyzed. Sufficient total intake of vegetables reduced the risk of breast cancer over the lifetime but not fruit intake (10). In addition, the results of this study are consistent with another comprehensive systematic review and meta-analysis conducted evaluate the evidence of the role of the dietary factor in the development of breast cancer among the Chinese women (20). Eight databases were searched and identified 33 case-control studies. Fruit and soy intakes were significantly correlated with a reduction of breast cancer but not for the fruit (20).

In regard to physical activities, the results varied and comparison was limited because of the use of different tools to measure the adequacy of physical activity (11, 20). Most of the previous studies reported no association between physical activity and the risk of breast cancer (11). This disparity might be because of the use of different tools to measure physical activity. For example, we evaluated duration and the effort involved in physical activity, while in a case-control study of 3.919 Japanese women, longer duration (> 300 min/wk) of physical activity was associated with a reduction in the risk of breast cancer (12).

Further, physical activity may reduce the bulk of fat accumulation in the body associated with increased risk of breast cancer. Further research using a unified measurement tool is needed at national and international levels.

The results also demonstrate that high total intake of calcium supplement is a risk factor for breast cancer, but again previous research reports are not consistent. Some studies support our results; for instance, a five-year longitudinal study reported an increase in the incidence of in situ breast cancer in a group of women who took calcium supplements (21). However, most of the studies found no association between total calcium intake and the occurrence of breast cancer (22, 23), while others reported a reduction in the risk (24). However, the current trend in the literature support that Calcium supplements have no association with breast cancer. A meta-analysis of sixteen randomized clinical trials that investigated this issue shows that adding calcium supplement for women did not increase their risk of having breast cancer (Relative Risk 1.01, 95% CI= 0.64 to 159, *P*=0.970). However, this variation in the results might be because of different interaction between human genes and surrounding environmental factors (2). Thus, re-investigating this result among Jordanian women is strongly recommended.

Practicing self-breast examination was presented as a risk factor; although it cannot be considered as a risk factor, self-examination helps in detecting breast cancer. As self-examination was associated with detecting more cases of breast cancer, women are recommended to practice it on a regular basis. On the other hand, some risk factors such as BMI, smoking and diabetes mellitus were found not to be associated with breast cancer. In a recent systematic review, decreased risk of breast cancer was observed among women with increased BMI (overweight and obese), while smoking was not associated with breast cancer (11). However, increased BMI, high fat intake, and smoking are linked to breast cancer. To resolve this disparity, a consensus on a standardized dietary-intake evaluation tool is required, together with and using a longitudinal perspective design in future studies.

### Strengths and Weakness

The results need to be understood in the light of the following limitations. First, selection and recall biases are inherent limitations of the case-control study design. Memorizing dietary habits, the type and amount of food taken are dependent on the subject’s ability to recall these details, which might differ from one person to another. In addition, patients in the case group may have become more aware of their dietary habits than those in the control group, because of their disease. Second, giving socially acceptable answers is a well-known limitation in the use of self-reporting questionnaires. Despite the limitations, this study sheds light on an area received little attention from Jordanian researchers, where most of the attention and care are directed to active cancer treatment.

### Implications for research and practice

First, further research using robust design is needed to either confirm or contradict the current results and thus improve breast cancer prevention measures and practice. Second, more efforts are required to develop and validate unified measurement tools for dietary intake and physical activity to enable comparability of studies results from different parts of the world. Finally, healthcare providers are advised to educate their patients in general and patients at high risk in particular about healthy lifestyle habits and its role in reducing the risk of breast cancer.

## Conclusion

Lifestyle risk factors were identified, and certain modifications to lifestyle are needed. Women’s awareness of these factors should be raised through appropriate channels, as a priority of the health authorities. Increasing the amount of high-quality research in this area remains one of the best ways to fight breast cancer, reducing its incidence and associated morbidities.

## Ethical considerations

Ethical issues (Including plagiarism, informed consent, misconduct, data fabrication and/or falsification, double publication and/or submission, redundancy, etc.) have been completely observed by the authors.
